# Role of MMP-9 in epithelial-mesenchymal transition of thyroid cancer

**DOI:** 10.1186/s12957-020-01958-w

**Published:** 2020-07-22

**Authors:** Yuanchun Li, Jing He, Feng Wang, Xin Wang, Fan Yang, Chunyang Zhao, Chunling Feng, Tiejun Li

**Affiliations:** 1 Department of General Surgery, The Second Affiliated Hospital of Qiqihar Medical University, No.37 Zhonghua West Road, Qiqihar, 161006 People’s Republic of China; 2 Department of Endocrinology and Metabolism, The Second Affiliated Hospital of Qiqihar Medical University, Qiqihar, 161006 People’s Republic of China; 3 Clinical Pathologic Diagnosis Center, Qiqihar Medical University, Qiqihar, 161006 People’s Republic of China; 4 Department of Clinical Medicine, Qiqihar Medical University, Qiqihar, 161006 People’s Republic of China

**Keywords:** Thyroid cancer, MMP-9, Epithelial-mesenchymal transition, Migration, Invasion

## Abstract

**Background:**

The purpose of this study is to explore the role and mechanism of MMP-9 in the EMT process of thyroid cancer (TC), so as to provide a basis for clinical exploration of invasion and metastasis process of TC, looking for biological markers of tumor metastasis and molecular intervention therapy.

**Methods:**

Western blot and RT-PCR were employed to detect the expression of MMP-9 in human normal thyroid cell line HT-ori3 and human TC cell lines IHH-4 (PTC), FTC-133, and 8505C. Expression levels of EMT-related markers: epithelial cell marker E-cadherin and stromal cell marker Vimentin in TGF-1-induced TC cell lines were detected by Western blot and RT-PCR, respectively. The effects of MMP-9 downregulation on cell invasion and metastasis were investigated by wound-healing assay and cell invasion experiment.

**Results:**

The protein and mRNA expression levels of MMP-9 in TC cell lines were increased compared with the human normal thyroid cell line HT-ori3. When TGF-β1 was added, the expression of EMT and Vimentin increased while the expression of E-cadherin decreased. Compared with the control group, the TC cells stably transfected with MMP-9 shRNA showed inhibited EMT, decreased Vimentin expression, and increased E-cadherin expression. The induction of TGF-β1 did not promote the occurrence of EMT in TC cells which were stably transformed with MMP-9 shRNA. The addition of TGF-β1 to TC cells increased the ability of the cells to migrate and invade. Compared with the control group, the migration and invasion ability of TC cells stably transfected with MMP-9 shRNA was significantly reduced, and the induction of TGF-β1 could not restore the migration and invasion ability of cells without MMP-9.

**Conclusions:**

In conclusion, we found that MMP-9 can be used as a biomarker for TC, which can promote the EMT process of TGF-β1 induced TC, and thus affecting the cell migration and invasion ability.

## Introduction

Thyroid cancer (TC) is one of the most common endocrine malignancies in the world, mainly derived from follicular cells [1]. In recent years, the incidence of TC in the world has increased year by year, ranking fifth in the incidence of malignant tumors [2]. Over the past decade, the incidence of TC of all pathological types has been on the rise. TC tends to metastasize and spread to the lymph nodes, sometimes to the lungs, bones, and brain [3]. Tumor metastasis is one of the most important causes of death in patients, with 90% of cancer patients dying from distant metastasis. Previous studies have shown that 3–5% of TC patients will develop distant metastasis [4], which always present a poor prognosis, with a 5-year survival rate of only 50%. Metastatic TC is aggressive and there is currently no curative treatment [5]. Therefore, it is very important and urgent to reveal the molecular mechanism involved in TC metastasis.

Epithelial tumor cells lose polarity and turn into mesenchymal cells. This is a process called epithelial-mesenchymal transition (EMT). Under normal physiological conditions, EMT plays a crucial role in embryonic development and tissue damage repair. In addition, EMT has been found to be an essential biological process for epithelial-derived malignant cell invasion, migration, and anti-apoptosis [6]. In the context of cancer, EMT has traditionally been viewed as a transcriptional regulatory process. During EMT, tumor cells can inhibit the transcription of epithelial genes (such as E-cadherin and keratin) and upregulate the transcription of mesenchymal genes (Vimentin and N-cadherin) to regulate multiple signals in the cancer microenvironment, so as to obtain the ability of migration and invasion. Transforming growth factor (TGF)-β signaling, which is upregulated in cancer development, usually triggers and drives the EMT process of cancer cells, and its role has been verified in a variety of human malignant tumors, including oral squamous cell carcinoma, hepatocellular carcinoma, pancreatic cancer, and esophageal cancer [7–10].

EMT is closely related to the invasion and metastasis of tumor cells. Metastasis is a major cause of death in most cancer-related patients, but the molecular mechanism of tumor cell proliferation remains unclear. Matrix metalloproteinases (MMPs) are zinc-dependent endopeptidases that can participate in proteolysis and can cleave several extracellular matrix (ECM) components and non-ECM molecules [11]. Its main function is to degrade various protein components of the extracellular matrix. MMP-1, MMP-8, and MMP-13 are the most secreted collagenases capable of destroying collagen I, II, III, V, and IX, as well as natural fibrillary collagen [12]. Recent research has shown that in addition to degrading various protein components of the extracellular matrix, some MMPs, like MMP-3, MMP-9, MMP-14, and MMP-2, can induce EMT or its related processes [13]. These proteases exert marked effects on tissue regeneration and organ development during ECM remodeling. According to previous studies, collagenase disorders can trigger a variety of pathological phenomena, including rheumatoid arthritis, chronic ulcers, and tumor invasion and metastasis [14]. Among them, MMP-9, also known as gelatinase B, is one of the key proteases for the degradation of extracellular matrix and basement membrane and plays a crucial part in the occurrence and development of malignant tumors, invasion, and metastasis, as well as angiogenesis [15]. As the most widely studied MMP, MMP-9 plays a crucial role in many biological processes, such as wound healing and tissue repair [16, 17]. It can regulate the structure of ECM by cutting and degrading multiple extracellular matrix proteins through protease hydrolysis [18]. Apart from that, MMP-9 is capable of specifically cutting the extracellular domain on the surface of certain cell proteins to release them from the plasma membrane, and some peptides can also be degraded by MMP-9 outside the cell [19]. The basement membrane contains collagen, such as type IV collagen, so it can be degraded by MMP-9 [20, 21]. During tumor development, the destruction of the basement membrane is often an essential step for tumors to achieve invasion and metastasis. Therefore, MMP-9 may act on the EMT process of tumors and can be an indispensable target for disease treatment. For example, studies have exhibited that MMP-9 is profoundly implicated in the invasion, metastasis, and angiogenesis of various tumors and can mediate the tumor microenvironment [22–26]. In view of the fact that the specific role of MMP-9 in the EMT process of TC cells has not been reported yet, it is very important and urgent to reveal the molecular mechanism involved in the metastasis of TC. MMP-9 is highly expressed in non-small cell lung cancer, cervical cancer, ovarian cancer, and pancreatic cancer and can be used as a biomarker in cancer prevention and diagnosis. But up to now, whether MMP-9 participates in the EMT process of TC induced by TGF-β1 remains poorly understood.

Therefore, this study mainly explores the role of MMP-9 in the EMT process of TC and its mechanism and further investigates the invasion and metastasis of TC for clinical purposes. At the same time, it also provides evidence for finding biomarkers of tumor metastasis and molecules for intervention therapy, further proposing important guidance for improving the survival of patients with TC.

## Materials and methods

### Cell culture

Human normal thyroid cell line HT-ori3 and human TC cell lines IHH-4 (PTC), FTC-133, 8505C were purchased from the Shanghai Institute of Cell Biology, Chinese Academy of Sciences. Cells were cultured on RPMI-1640 (Hyclone, USA) complete medium containing 10% fetal bovine serum (Hyclone, USA), 1% penicillin, and 1% streptomycin (Invitrogen, USA) in a constant temperature incubator at 37 °C and 5%CO_2_. When the cell fusion degree reached 90%, the culture medium was discarded, and the cells were rinsed with PBS buffer and then added with trypsin to digest the cells. After the cells were detached, the complete medium was added, and the cells were pipetted down and centrifuged in a centrifuge tube. Upon centrifugation, the supernatant was discarded and the cells were processed for subculture.

### shRNA stable transfection

The target sequence of the MMP-9 shRNA is 5′-GCCTGCAACGTGAACATCTTCGACGCCAT-3′ (GenePharma Co., Ltd, Shanghai, China). One day before transfection, the cells were digested with trypsin and then inoculated into a new sterile cell culture dish (the specification of the dish was selected according to the specific experimental requirements). The next day, cell density was observed (preferably around 80%) and an appropriate amount of shRNA was transfected. Then, the cells were cultured in a constant temperature incubator at 37 °C and 5% CO_2_ for 24 h and then cultivated in a complete medium containing the eukaryotic screening drug G418 (Invitrogen, USA). The fresh medium was replaced every other day. After a week or so, when a large number of untransfected shRNA cells died, and monoclonal began to appear in successfully transfected cells, the screening drug concentration was halved and culture continued. Cell lines stably expressing exogenous genes can be obtained in about 2–3 weeks.

### Real-time PCR

Total RNA of cells was extracted by Trizol (Invitrogen, USA) method. Reverse transcription was performed according to the instructions of the TIANGEN Kit (QuantScript RT Kit, KR103), and real-time PCR was carried out by referring to the Thermo Kit instructions (PowerUp™ SYBR™ Green Master Mix, A25780). See Table 1 for the primer sequences of the target gene and internal reference gene.

**Table 1:** The primer sequences of the target gene and internal reference gene

	Forward	Reverse
MMP9	5′-GGGACGCAGACATCGTCATC-3′	5′-TCGTCATCGTCGAAATGGGC-3′
E-cadherin	5′-TGCTCTTCCAGGAACCTCTGTG-3′	5′-GGTGACCACACTGATGACTCCTG-3′
vimentin	5′-GGGACCTCTACGAGGAGGAG-3′	5′-CGCATTGTCAACATCCTGTC-3′
β-actin	5′-TTAGTTGCGTTACACCCTTTC-3′	5′-ACCTTCACCGTTCCAGTTT-3′

The amplification conditions were as follows: pre-denaturation at 95 °C for 10 min, denaturation at 95 °C for 10 s, annealing at 2 °C for 20 s, and extension at 72 °C for 10 s. In order to verify the specificity of the amplification product, a solution curve was added after PCR. The solution curve had a single peak for specific amplification and a bimodal or multi-peak for non-specific amplification. All tests were performed on 96-well plates, with three duplicate wells for each sample, and internal parameters for the detection of β-actin were set for relative quantification by 2^-ΔΔCT^ method.

### Western blot

Cells were lysed with a cell lysate to prepare a cell lysate. Protein concentration was determined using a BCA kit (KeyGen Biotechnology, Co., Ltd., Jiangsu, China, KGP902). The extracted protein was sampled at about 50 μg per well and was separated by 10% SDS-PAGE gel electrophoresis before transferring to the PVDF membrane. Next, the PVDF membrane was sealed with a TBST buffer containing 5% skim milk powder at room temperature for 1 h and incubated with the primary antibody in a refrigerator at 4 °C overnight. After cleaning with TBST for 3 times, the membrane was incubated at room temperature for 1 h with the secondary antibody, which was then washed away by TBST and finally the membrane was displayed by ECL luminescence liquefaction. See Table 2 for the name, item number, and working concentration of the antibody used in the experiment.

**Table 2:** The name, item number, and working concentration of the antibody used in the experiment

Antibody name	Company	Article number	Working concentration
β-actin	ZSGB-Bio, Beijing, China	TA-09	1:1000
E-cadherin	Santa Cruz, USA	sc-8426	1:200
Vimentin	CST, USA	5741	1:1000
Goat anti-mouse IgG	Bioss, Beijing, China	bs-0296G	1:3000
Goat anti-rabbit IgG	Bioss, Beijing, China	bs-0295G	1:3000

ImageJ software was employed to analyze the gray value of the target protein, and β-actin was set as the internal reference for statistical analysis.

### Wound-healing assay

Before seeding the cells, three horizontal lines with the same spacing were drawn with a pen at the bottom of the 6-well plates, running through each well of the plate. Approximately, 1 × 10^6^ cells were seeded in each well, and the degree of cell confluence should be 100% after overnight. The next day, a micropipette tip was applied to create a wound in the direction of a ruler, perpendicular to the horizontal line at the bottom, and the gun tip should be vertical. The cells were then rinsed with PBS and added to fresh complete medium. After that, the well plate was cultured in a constant temperature incubator of 37 °C and 5%CO_2_. The culture plate was taken out At 0 h, 6 h, 12 h, and 24 h, at which time points, pictures were taken under the microscope at the same visual field. The mean distance between cells was calculated by ImageJ software.

### Cell invasion experiment

Diluted with double medium-free, 50 μg/ml Matrigel gel (BD, USA) was taken to prepare base adhesive. After adding 100 μl base adhesive to each well in the Insert chamber (Corning, USA) (8 μm), the chamber was placed in a 24-well plate and dried at 37 °C to form an artificial basement membrane. Before inoculating the cells, the prepared basement membrane was incubated with 100 μl/well double non-medium at room temperature for 40 min for hydration treatment. Each chamber was inoculated with 20000 cells. According to the needs of the experiment, 200-μl cell suspension was inoculated in each well, and the medium containing 1% fetal bovine serum was applied in the chamber, while 600 μl complete medium containing 10% fetal bovine serum was added to the 24-well plate in the lower chamber. After inoculation, it was cultured in a 5%CO_2_ incubator at 37 °C for 48 h, followed by the removal of the medium and a triple meticulous rinse of the chamber with PBS at 100 μl/well. Then, the 24-well plate was fixed with 600 μl of 4% paraformaldehyde at room temperature for 30 min, washed with PBS solution for 3 times, and then stained with 600-μl crystal violet per well for 5 min. After cleaning and drying, photos were taken at randomly selected visual fields with a microscope, and at least 3 pictures of each well were obtained for statistical analysis.

### Statistical analysis

All the collected data were processed by GraphPad Prims 6.0 software, and the statistical data was expressed as the mean ± standard deviation. Inter-group comparison of means was conducted by the *t* test, while multi-group comparison of means was performed by analysis of variance (ANOVA). *P* < 0.05 was considered to be statistically significant.

## Results

### Specific high expression of MMP-9 in TC cell lines

**Fig. 1:**
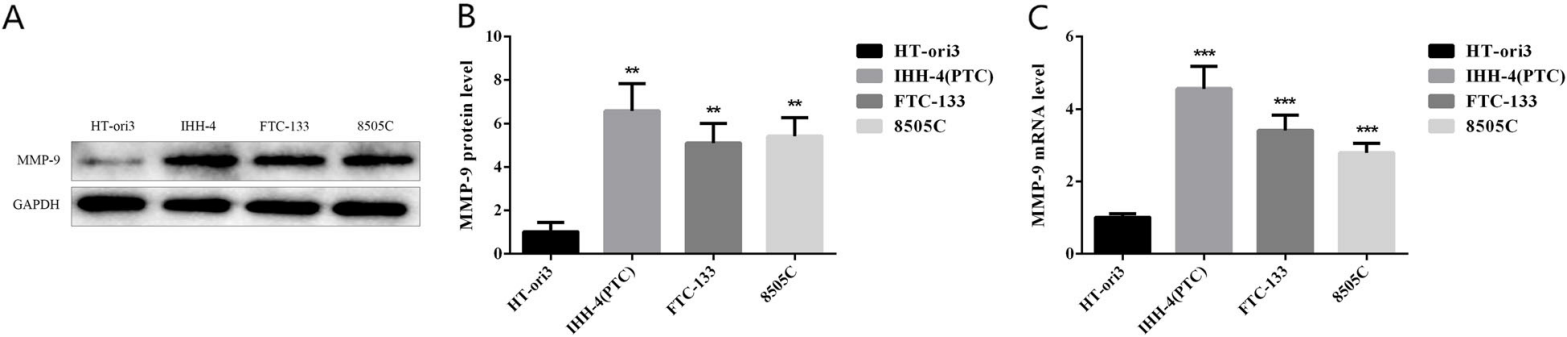
MMP-9 expression levels in normal human thyroid and TC cell lines. **a**, **b** MMP-9 protein expression levels in HT-ori3, IHH-4 (PTC), FTC-133, and 8505C cell lines and statistical results of gray values of the bands. **c** MMP-9 mRNA expression levels in T-ori3, IHH-4 (PTC), FTC-133, and 8505C cell lines, ** indicates *P* < 0.01, and *** indicated *P* < 0.001

**Fig. 2:**
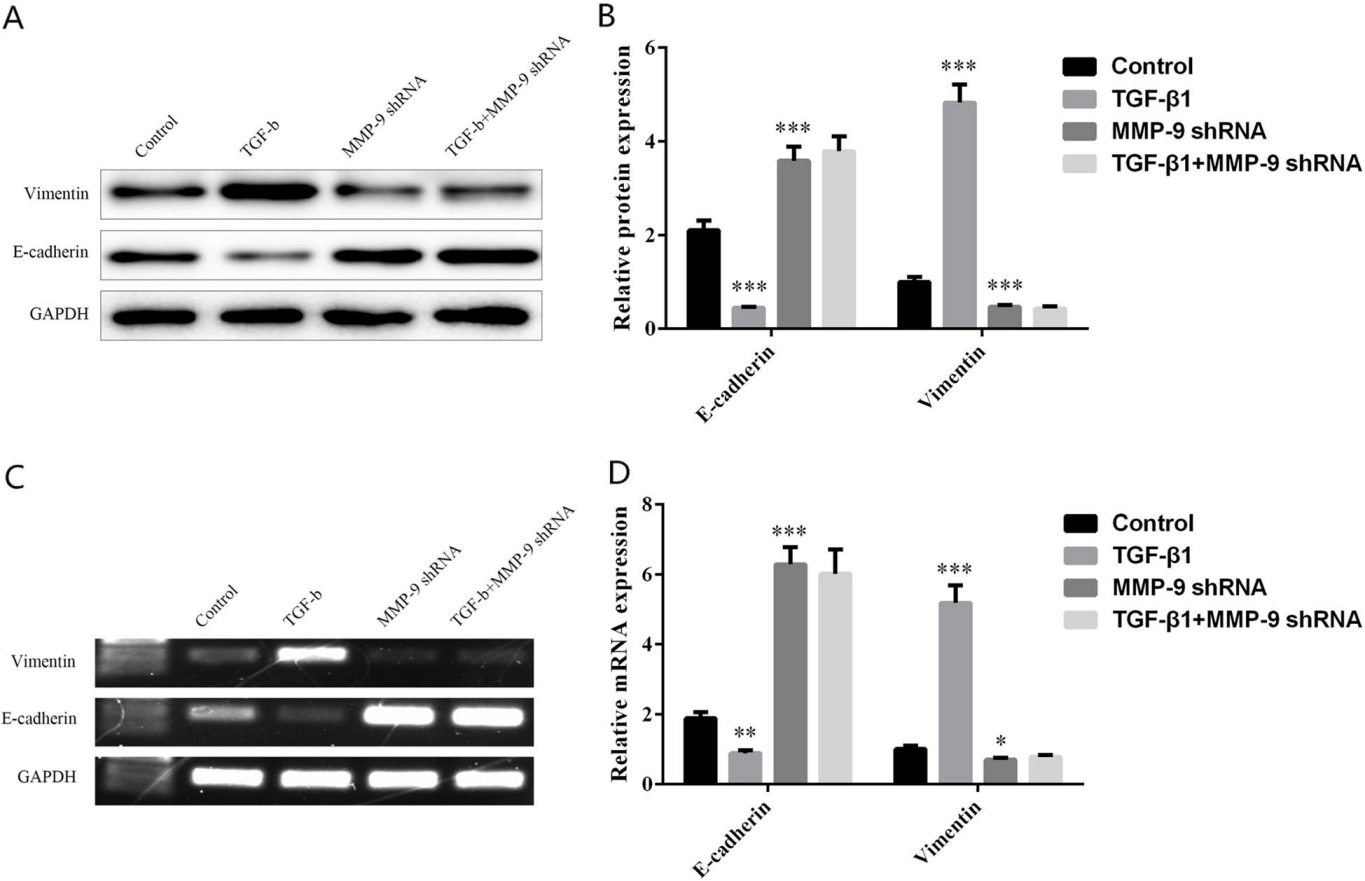
Effects of MMP-9 on the expression level of EMT-related proteins in TC cells. The protein levels (**a**, **b**) and mRNA levels (**c**, **d**) of epithelial cell marker E-cadherin and stromal cell marker Vimentin in TC cell lines transfected with TGF-β1 MMP-9 shRNA, and TGF-β1 + MMP-9 shRNA. *Indicates *P* < 0.05, ** indicates *P* < 0.01, and *** indicates *P* < 0.001

According to the results of Western blot and RT-PCR, the protein expression level (Fig. 1) and mRNA expression level (Fig. 2) of MMP-9 in TC cell lines IHH-4 (PTC), FTC-133, and 8505C were elevated compared with human normal thyroid cell line HT-ori3. The relative gray value of protein expression levels of MMP-9 in normal thyroid cells compared with TC cell lines were 1.01 ± 0.43 vs 6.59 ± 1.24, 5.10 ± 0.91, and 5.42 ± 0.86 (*P* = 0.0081, *P* = 0.0022, *P* = 0.0030, respectively). While the mRNA expression levels of MMP-9 in normal thyroid cells compared with TC cell lines were 1.01 ± 0.09 vs 4.56 ± 0.61, 3.41 ± 0.42, and 2.79 ± 0.26, respectively (*P* = 0.0006, *P* = 0.0007, *P* = 0.0004, respectively).

### MMP-9 altered the changes of EMT-related proteins in TC cells induced by TGF-β1

Western blot and RT-PCR experiments demonstrated that compared with control cells, the addition of TGF-β1 could promote cell EMT, increase the expression of Vimentin, and decrease the expression of E-cadherin. As to TC cells stably transfected with MMP-9 shRNA, they presented suppressed EMT progress of EMT, reduced Vimentin expression, and elevated E-cadherin expression than the control group. Later, it was found that adding TGF-β1 to TC cells stably transfected with MMP-9 shRNA did not promote the occurrence of EMT in the cells (Fig. 2a, c). The protein expression levels of E-cadherin in the control group, TGF-β1 group, MMP-9 shRNA group, and TGF-β1 + MMP-9 shRNA group were 2.11 ± 0.20, 0.45 ± 0.02, 3.59 ± 0.31, and 3.79 ± 0.32, and those of Vimentin were 1.00 ± 0.11, 4.83 ± 0.38, 0.47 ± 0.04, and 0.43 ± 0.05, respectively (Fig. 2b). While the mRNA levels of E-cadherin in the control group, TGF-β1 group, MMP-9 shRNA group, and TGF-β1 + MMP-9 shRNA group were 1.89 ± 0.18, 0.89 ± 0.08, 6.30 ± 0.49, and 6.02 ± 0.70, respectively, and the mRNA levels of Vimentin were 1.01 ± 0.10, 5.19 ± 0.50, 0.70 ± 0.06, and 0.78 ± 0.06, respectively (Fig. 2d).

### Downregulation of MMP-9 expression inhibited TGF-β1-induced migration of TC cells

**Fig. 3:**
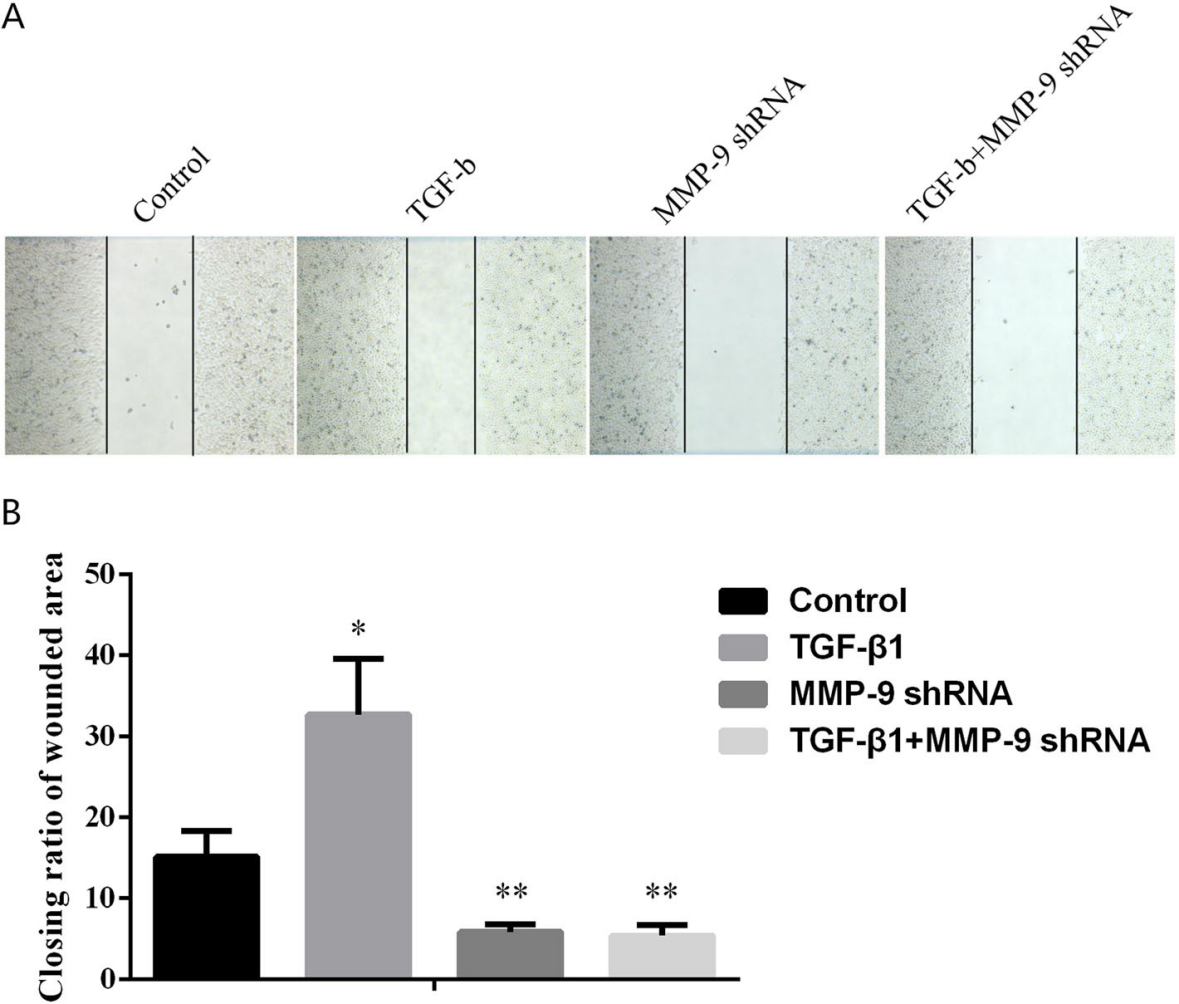
Effects of MMP-9 on the migration of TC cells. **a**, **b** After adding TGF-β1, MMP-9 shRNA, and TGF-β1+ MMP-9 shRNA to TC cell lines, the wound healing ability of TC cells was observed. *Indicated *P* < 0.05, and ** indicated *P* < 0.01

The relative area of cell migration in the control group, TGF-β1 group, MMP-9 shRNA group, and TGF-β1 + MMP-9 shRNA group was 15.12 ± 3.20, 32.69 ± 6.83, 5.85 ± 0.92, and 5.39 ± 1.29, respectively. The above data suggested a statistical difference between the control group and the TGF-β1 group (*P* = 0.037), and between the control group and the MMP-9 shRNA group (*P* = 0.009), while no significant difference was observed between the MMP-9 shRNA group and TGF-β1 + MMP-9 shRNA group. Therefore, it could be concluded that the downregulation of MMP-9 expression was able to inhibit the occurrence of EMT and cell migration of TC cells induced by TGF-β1. See Fig. 3.

### Downregulation of MMP-9 expression inhibited TGF-β1-induced invasion of TC cells

The relative numbers of invasive cells in the control group, TGF-β1 group, MMP-9 shRNA group, and TGF-β1 + MMP-9 shRNA group were 65.11 ± 8.42, 122.38 ± 16.43, 26.81 ± 6.76, and 28.37 ± 9.41, respectively. The above data suggested that there was a statistical difference between the control group and the TGF-β1 group (*P* = 0.0037), and between the control group and the MMP-9 shRNA group (*P* = 0.0019), while no significant difference was observed between the MMP-9 shRNA group and TGF-β1 + MMP-9 shRNA group. Thus, we could conclude that the downregulation of MMP-9 was capable of inhibiting the development of EMT and cell invasion of TC cells induced by TGF-β1.

## Discussion

Many studies have reported an association between elevated MMP-9 levels and increased rates of cancer metastasis and poor clinical outcomes. In this paper, we explored the effect of MMP-9 on the development of thyroid cancer (TC) and verified that MMP-9 could promote the EMT process of TC induced by TGF-β1. Our study revealed that the high expression of MMP-9 may be one of the factors influencing the high aggressiveness and poor prognosis of TC.

TC is a common endocrine malignancy in the world [27], and its incidence grows year by year [1]. Tumor metastasis is one of the leading causes of death in patients, with 90% of cancer patients dying from distant metastasis of malignant solid tumors. Previous studies have shown that 3–5% of TC patients will develop distant metastasis [4], and such patients have a poor prognosis, with a 5-year survival rate of only 50%. Metastatic TC is aggressive; however, at present, there is no curative treatment [5]. Therefore, it is high time to elucidate the molecular mechanism involved in TC metastasis. EMT is bound up with the invasion and metastasis of tumor cells. Metastasis is a major cause of death in most cancer-related patients, but the molecular mechanism of tumor cell proliferation remains a subject of investigation. EMT is an important model of metastasis and diffusion, in which epithelial cells lose their adhesion properties and gain fibroblast-like morphology and increase motility. In the development of cancer, it has been suggested that EMT plays an important role in invasion, blood-borne transmission, and cell acquisition of chemical resistance [6]. Tumor metastasis is a series of complex biological processes, involving many proteins and molecules, which mainly include the following three steps: the decrease of adhesion, the degradation of matrix, and the increase of mobility. The matrix metalloproteinases (MMPs) family is the main protease family that degrades ECM and produces marked effects on the EMT process of tumors. MMP is a family of zinc-dependent endopeptidases with more than 20 different family members [28], which can be divided into several categories, such as gelatinase, collagenase, and stromelysin [29]. MMP-9, which plays an important role in cancer cell invasion and tumor metastasis, is one of the most important members of the MMP family.

It is well established that MMP-9 is profoundly implicated in the invasion, metastasis, and angiogenesis of various tumors and can mediate the tumor microenvironment [22–26]. In view of the fact that the specific role of MMP-9 in the EMT process of TC cells has not been reported yet, it is of great significance and urgency to reveal the molecular mechanism involved in the metastasis of TC.

What is more, MMP-9 has been found to be significantly overexpressed in TC compared with adjacent non-tumor tissues [30]. In this study, we also verified that compared with human normal thyroid cell line HT-ori3, the protein expression level of MMP-9 in TC cell line (Fig. 1a), and mRNA expression level (Fig. 1c) were increased, which was consistent with the previous trend in tissue samples, suggesting that MMP-9 can be used as a potential biomarker of cancer, and its role as a therapeutic target in the fields of tumor diagnosis, therapeutic effect monitoring, and disease progression monitoring.

**Fig. 4:**
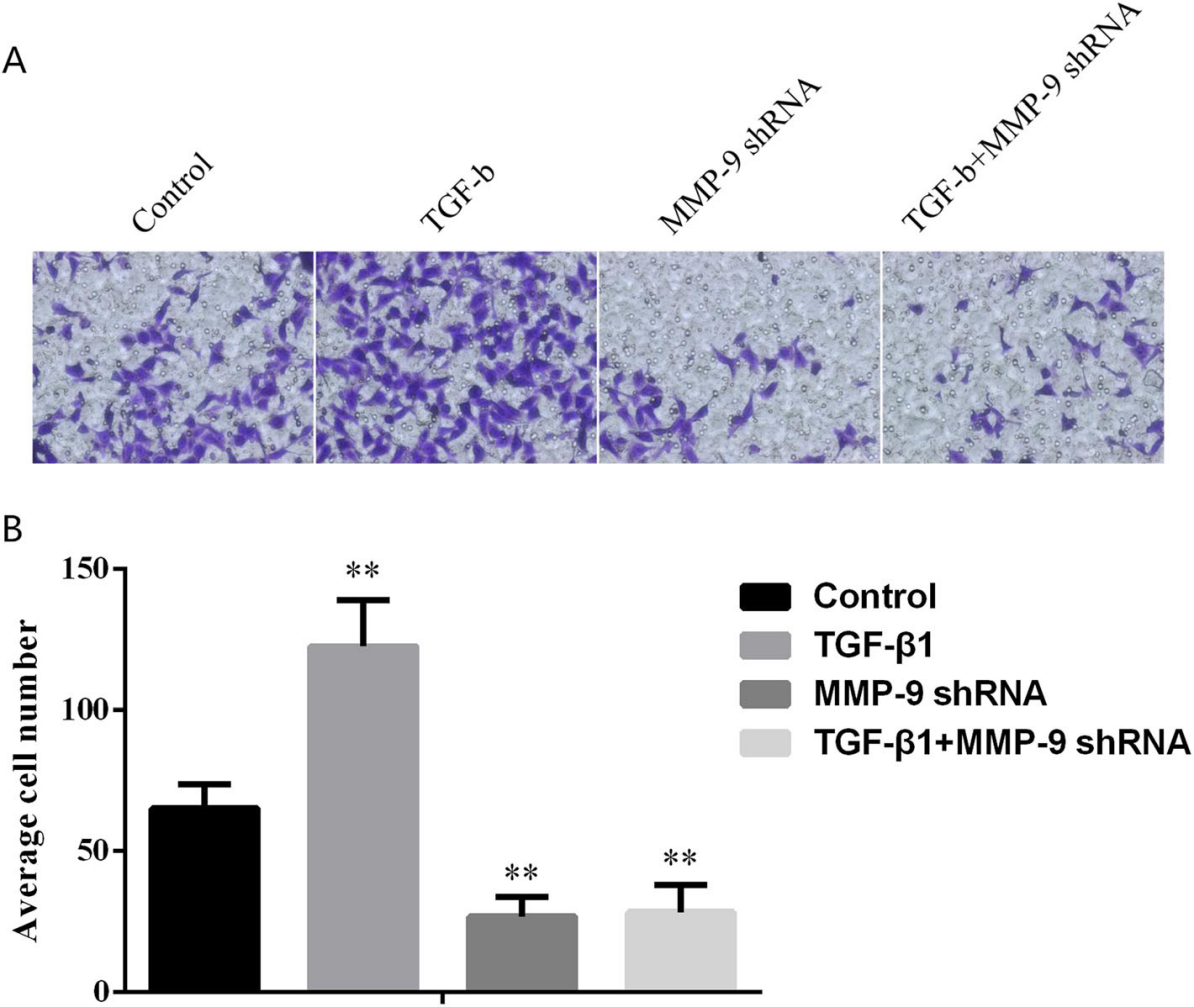
Effects of MMP-9 on the invasion of TC cells. **a**, **b** In TC cell lines, the invasion ability of TC cell lines induced by MMP-9 shRNA and TGF-β1 was observed (**a** is the photo, **b** shows the three statistical results). *Indicated *P* < 0.05 and ** indicated *P* < 0.01

Previous studies have evaluated the expression level of MMP-9 in human TC through immunohistochemical experiments and revealed that the level of MMP-9 in more invasive tissues was significantly higher than that in other tissues [31]. In this study, we carried out experiments in TC cells. It turned out that compared with the control cells, the addition of TGF-β1 could increase the expression of EMT and Vimentin and decrease the expression of E-cadherin in TC cells, with significant differences. When compared with the control group, the TC cells stably transfected with MMP-9 shRNA brought inhibited process of EMT, decreased Vimentin, and increased E-cadherin expression levels. Later, it was noticed that the induction of TGF-β1 made no difference in promoting the occurrence of EMT in TC cells which were stably transformed with MMP-9 shRNA (Fig. 2). The experimental results suggested that the high expression of MMP-9 in tumor cells promoted the occurrence of EMT by increasing the expression of Vimentin and reducing the expression of E-cadherin. What is more, we found that the addition of TGF-β1 to TC cells increased the ability of the cells to migrate and invade. Compared with the control group, the migration and invasion ability of TC cells stably transfected with MMP-9 decreased significantly, and the addition of TGF-β1 could not restore the migration and invasion ability of cells without MMP-9 (Figs. 3 and 4), suggesting that MMP-9 could enhance the EMT process of TGF-β1 induced TC, and thus affect cell migration and invasion. Moreover, after the knockout of MMP-9 and induction of TGF-β1, the cells would not produce EMT, indicating that MMP-9 is indispensable in the process of TGF-β1-induced EMT. MMP-9 plays a vital role in the degradation of type IV collagen (the main component of the basement membrane), thereby promoting tumor invasion. Its abnormal secretion will damage the basement membrane and cause tumor invasion and metastasis. In addition, it is well known that highly expressed MMP-9 will induce tumors to be more aggressive. In this study, we further explained the reason behind the enhanced invasion of tumors caused by the high expression of MMP-9. It may be that it affected the EMT process induced by TGF-β1 and then promoted the invasion and metastasis of TC. Furthermore, we reverse verified the MMP-9 knockout in TC cells, which also confirmed our hypothesis. The EMT process is under the control of various factors, among which TGF-β counts the most. Interfering with the expression of TGF-β or regulating its downstream signaling molecules can affect the EMT process induced by TGF-β. This study has confirmed that MMP-9 can affect the EMT process of thyroid cells and thus promote the invasion and migration of cells; however, no further study has been conducted on whether MMP-9 has a regulatory effect on molecules in the TGF-β signaling pathway. Therefore, in our follow-up research, we will focus on whether the molecules in the MMP-9-TGF-β signaling pathway can interact with each other, thus confirming that MMP-9 regulates the cellular EMT process by affecting TGF-β, providing more possibilities for clinical targeted therapy.

In conclusion, MMP-9 is specifically overexpressed in TC and can be used as a biomarker for TC. It can promote the EMT process in TGF-β1 induced TC, which in turn affects the ability of cells to migrate and invade. This study advances a basis for clinical exploration of the process of invasion and metastasis of TC, provides clues for finding biological markers of tumor metastasis, and plays an important guiding role in further improving the survival time of patients with TC.

## Data Availability

The datasets used and/or analyzed during the present study are available from the corresponding author on reasonable request.
